# On a desmitracheate “micronetine” *Nippononeta alpina* (Li & Zhu, 1993), comb. n. (Araneae, Linyphiidae)

**DOI:** 10.3897/zookeys.645.10685

**Published:** 2017-01-13

**Authors:** Mengdie Bao, Zishang Bai, Lihong Tu

**Affiliations:** 1 College of Life Sciences, Capital Normal University, Xisanhuanbeilu Str. 105, Haidian Dist. Beijing, 100048, P. R. China; 2 The High School Affiliated to Renmin University of China, Beijing, 100080

**Keywords:** Epigynal scape, genital morphology, phylogenetic placement, tracheal system

## Abstract

The phylogenetic analyses based on molecular data demonstrate that all “micronetine” species of a desmitracheate system form a monophyly. *Macrargus* Dahl, 1886 is a “micronetine” genus, the species of which have a haplotracheate system in general, while *Macrargus alpinus* Li & Zhu, 1993 was found to have a desmitracheate system; this makes its generic placement problematic. According to the results of phylogenetic analysis, we transfer *Macrargus alpinus* to another genus as *Nippononeta alpina* (Li & Zhu, 1993), **comb. n**., and provide a redescription of its genital characters and somatic features. Comparisons with other “micronetine” species with a desmitracheate system are provided. Putative synapomorphies for *Nippononeta*, the clade *Nippononeta* + *Agyneta*, and for the “desmitracheate micronetines” clade, as well as their relationship with *Helophora*, are provided and discussed.

## Introduction

Linyphiidae Blackwall, 1859 is a species-rich family of spiders which has species-specific genitalia but more conservative somatic features in general. The tracheate system in linyphiids consists of two pairs of trachea; “desmitracheate” and “haplotracheate” are the two terms referring to the two main tracheal conditions having the median pair highly branched and unbranched, respectively (Blest 1976, Millidge 1984). The seven subfamilies currently accepted in Linyphiidae are delimited largely based on genital characters, except for Erigoninae Emerton, 1882, which was originally defined by having a simple type male palp (Merrett 1963, Millidge 1977), and then redefined on the basis of its desmitracheate system (Blest 1976, Millidge 1984). Such a classification for “erigonines” has long been puzzled by the observations that some “erigonine” species have genitalia of simple type, but possess haplotracheate systems (Hormiga 2000, Miller and Hormiga 2004), while some “micronetine” species have genitalia of complex type, but possess desmitracheate systems (Millidge 1984, Dupérré 2013, Yan et al. 2015). Results of phylogenetic analyses based on molecular data recovered the monophyly of the Linyphiidae, in which the taxa of “micronetines” and “erigonines” nest (Arnedo et al. 2009, Wang et al. 2015); however, all “micronetine” species with a desmitracheate system form a monophyly (addressed as “desmitracheate micronetines” clade hereafter). Ancestral state reconstructions indicate that both tracheal features and genital characters used in subfamily classification are homoplastic. Furthermore, conversions between haplotracheate and desmitracheate conditions have taken place multiple times, and usually at the generic level. Until now, no evidence indicated that both tracheal conditions co-occur among congeneric species.

*Macrargus* Dahl, 1886 is a typical “micronetine” genus with a haplotracheate system (Blest 1976). However, *Macrargus alpinus* Li & Zhu, 1993, occurring in China, was found to have a desmitracheate system, different from its congeners. The “desmitracheate micronetines” clade resulting from phylogenetic analyses is distantly related to *Macrargus* (Wang et al. 2015). Some putative synapomorphies for the desmitracheate “micronetine” genus *Nippononeta* Eskov, 1992 and for the clade *Nippononeta*+*Agyneta* proposed by Yan et al. (2015) can also be found in *Macrargus alpinus*, yet none of them is present in other *Macrargus* species. This implies that the generic placement of *Macrargus alpinus* is questionable.

To test the phylogenetic placement of *Macrargus alpinus* and its relationships with other desmitracheate “micronetines”, we added the newly sequenced DNA sequence data of *Macrargus alpinus* into the dataset of Wang et al. (2015). In the present study, we propose a new generic placement for *Macrargus alpinus* based on the result of phylogenetic analysis of the new dataset. We present a redescription of *Macrargus alpinus* and comparisons with closely related groups. Putative synapomorphies for *Nippononeta* and the desmitracheate “micronetine” groups proposed by Yan et al. (2015) are revised for further studies.

## Materials and methods

### Phylogenetic analysis

Two mitochondrial genes, cytochrome c oxidase subunit I (CO1) and 16S rRNA (16S), and two nuclear genes, 18S rRNA (18S), and 28S rRNA (28S) were amplified and sequenced for *Macrargus alpinus* and added to the dataset of Wang et al. (2015) to test its placement in Linyphiidae. Given that the primary analysis resulted in *Macrargus alpinus* as a sister group to *Nippononeta coreana*, the four genes data of one additional *Nippononeta* species was downloaded from GenBank and added to test the monophyly of the genus *Nippononeta* and its relationship with *Macrargus alpinus*. Based on the dataset of Wang et al. (2015), a total of 132 taxa was included in our matrix: 130 linyphiid species including the type species of *Macrargus*, seven species from four genera as representatives of desmitracheate “micronetines”, and two representative species of *Nippononeta*. Most outgroup taxa of Wang et al. (2015) were removed; only two representatives of Pimoidae, the sister group of Linyphiidae, were included as outgroups to root the tree.

Molecular protocols for amplification and sequencing follow that of Wang et al. (2015). Taxa sampled and sequence accession numbers are presented in Wang et al. (2015), and those of the two new taxa are presented in Suppl. material 1. Every sequence was first aligned using CLUSTAL X version 1.81 (Thompson et al. 1997) independently, and then the sequences of four genes were concatenated by MESQUITE (Version 2.75, Maddison and Maddison 2009). The genes were unpartitioned. The gaps were considered as missing data. Maximum Likelihood analysis of the concatenated dataset was conducted by RAxML v. 7.2.7 as implemented on the Cipres Gateway (Miller et al. 2010), using GTR+I+R model, which was the best fitting model for the matrix by JModeltest examination. Bootstrap analysis was obtained with 1000 replicates to assess nodal support.

### Morphological methods

Specimens were examined and illustrated by using a Leica M205A stereomicroscope and a Leica DM5500B compound microscope. The male palp and female epigynum were examined after they were dissected from the body. The embolic division was excised by breaking the membranous column connecting between the suprategulum and radix. For microscopic examination and illustration, the male palp and epigynum were cleared in methyl salicylate. Illustrations were made using a drawing tube. Scanning Electron Microscopy (SEM) images were taken by using a LEO 1430VP at the Department of Biological Sciences at George Washington University. For SEM examination the specimens were prepared following Álvarez-Padilla and Hormiga (2008). SEM images of the embolic division taken from the right palp were mirrored to match those taken from the left palp. All specimens examined here are deposited in the Institute of Zoology, Chinese Academy of Sciences, Beijing, China (IZCAS), and the College of Life Sciences, Capital Normal University, China (CNU). Terminology for the genital and somatic characters follows Hormiga (2000), Tu and Hormiga (2010, 2011), Saaristo and Tanasevitch (1996) and Wang et al. (2015).

## Results

**Figure 1.**
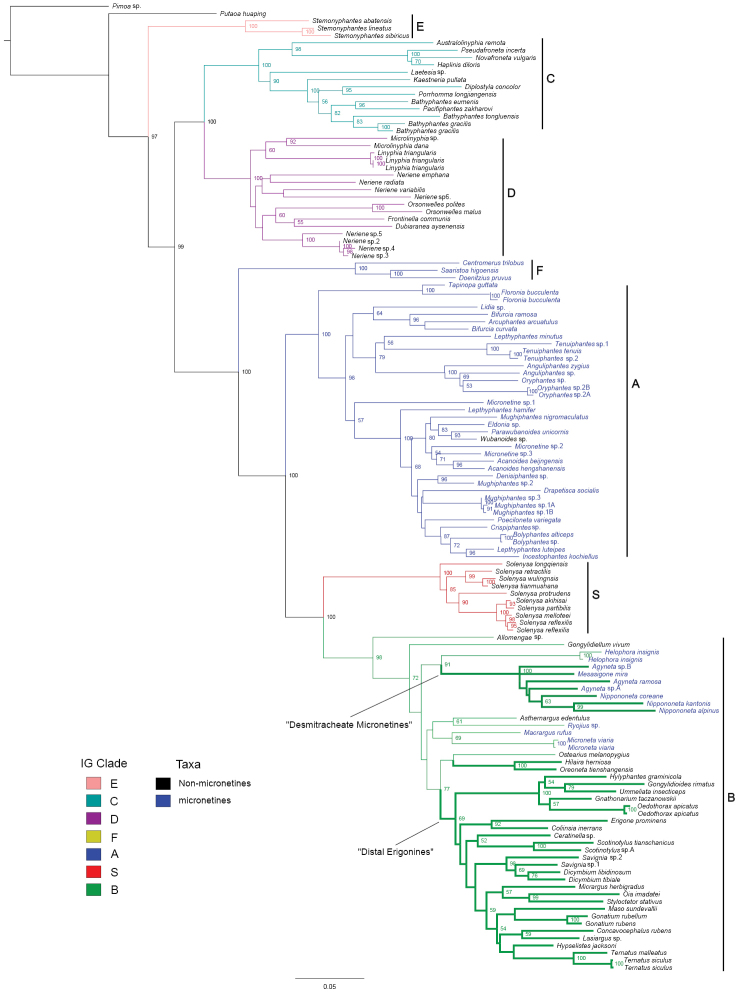
Linyphiid phylogeny resulting from the Maximum Likelihood analysis based on DNA sequence data. Numbers at nodes indicate bootstrap support above 50%. Branches in color represent seven robustly supported main clades within Linyphiidae. Branches in bold indicate the desmitracheate type. Taxa in blue are currently placed in Micronetinae.

With the data on *Macrargus alpinus* and an additional *Nippononeta* species added to their dataset, the Maximum Likelihood analysis recovered the general topology of Wang et al. (2015): the monophyly of Linyphiidae, its sister relationship with Pimoidae, the seven main clades and relationships among them within linyphiids (all with bootstrap > 95%), with variations on the placement of some weakly supported lineages within these clades (Fig. 1). “Micronetines” remain paraphyletic, forming clades A and F, as well as some basal lineages of clade B, nesting with “erigonines”. Three desmitracheate clades are included within clade B. Besides the “distal erigonines” clade (bootstrap = 69%) and one other “desmitracheate erigonines” lineage (bootstrap = 100%), all those “micronetine” species of a desmitracheate system form a well-supported clade, the “desmitracheate micronetines” clade (bootstrap = 100%), sister to the *Helophora* clade (bootstrap = 91%). *Macrargus rufus* and *Microneta viaria* form one of the “haplotracheate micronetines” lineages within clade B (bootstrap = 69%). Meanwhile, *Macrargus alpinus* falls into the *Nippononeta* clade (bootstrap = 63%), sister to *Nippononeta kantonis* (bootstrap = 99%), but distantly related to *Macrargus rufus*. The monophyly of *Agyneta* and its relationship with the *Nippononeta* clade remain unresolved.

### Taxonomy

#### Linyphiidae Blackwall, 1859

##### Nippononeta

Taxon classificationAnimaliaAraneaeLinyphiidae

Eskov, 1992

###### Type species.

*Nippononeta kurilensis* Eskov, 1992.

##### Nippononeta alpina

Taxon classificationAnimaliaAraneaeLinyphiidae

(Li & Zhu, 1993) comb. n.

Macrargus alpinus Li & Zhu, in Song et al. 1993: 863, f. 21A–I (D♂♀); Li et al. 1994: 81, f. 31–33 (♀); Li and Zhu 1995: 41, f. 2a–i (♂♀); Song et al. 1999: 186, f. 104D, G, J (♂♀).

###### Type material examined.

♂ holotype (IZCAS), China, Hubei Province, Shennongjia Natrual Conservation, Panlong County, 26 June 1986; 1♂ and 2♀ paratypes (IZCAS), same data as the holotype.

###### Additional material examined.

4♂ and 4♀(CNU), China, Sichuan Province, Lushan County, Fenghuo town, Sanyou village, 7 July 2004, L. Tu leg; 5♂ and 4♀(CNU), China, Sichuan Province, Tianquan County, Mt. Erlangshan Natural Forest Park, 8 July 2004, L. Tu leg; 3♂ and 4♀(CNU), China, Zhejiang Province, Mt. Yandangshan, 28°35.78’ N, 121°04.30’ E, alt. ca 420m, 15 Aug. 2010, F. Wang leg.

###### Diagnosis.

**Figure 2.**
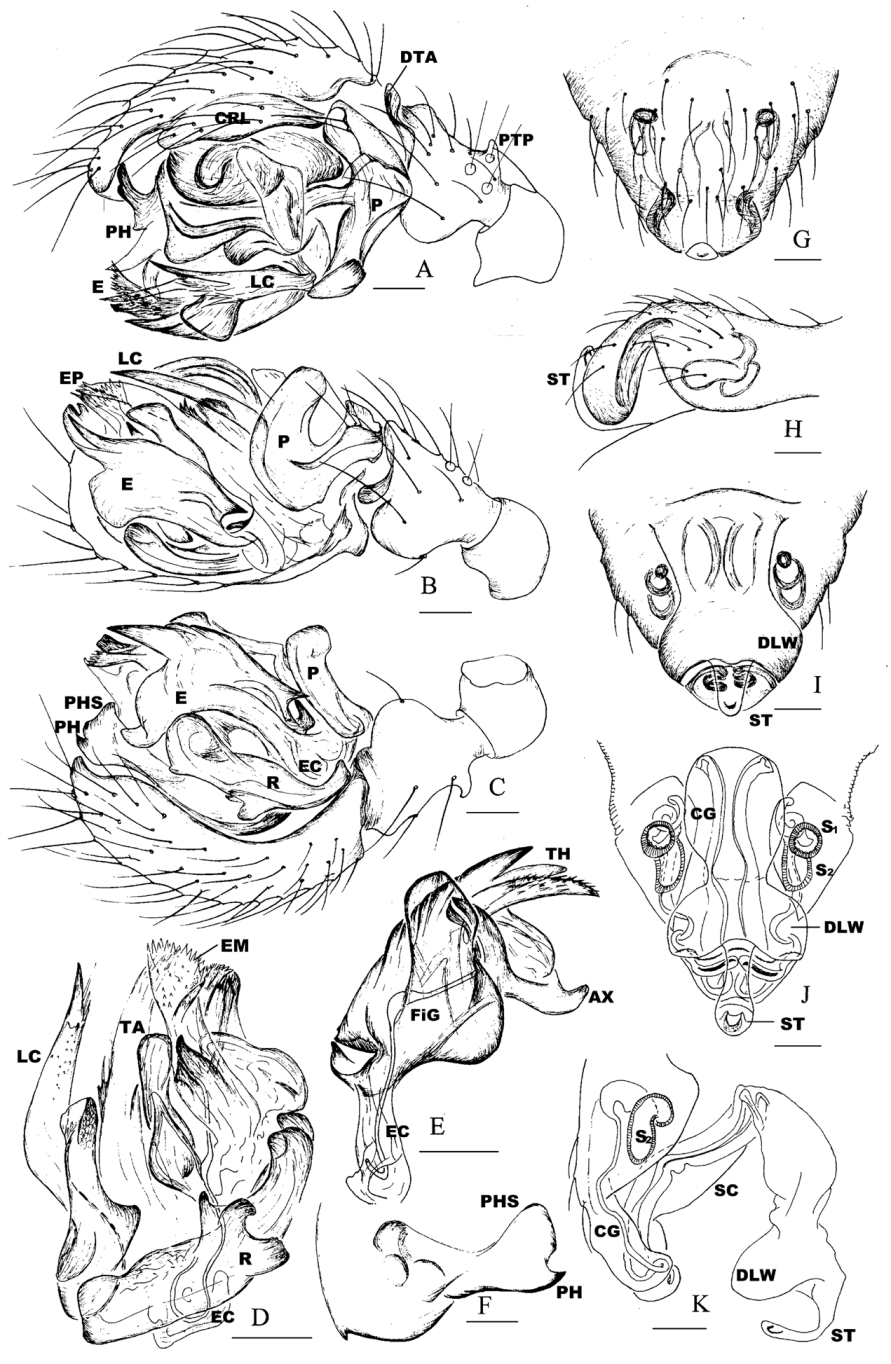
*Nippononeta alpina* comb. n. (**A–F**) male palp **A** retrolateral **B** ventral **C** prolateral **D** embolic division **E** embolus **F** distal suprategular apophysis (**G–K**) epigynum **G** ventral **H** lateral **I** dorsal **J** dorsal, cleared **K** lateral, cleared. Abbreviations: AX apex of embolus; CG copulatory groove; CRL cymbial retrolateral lobe; DLW lateral wing on distal part of scape; DTA distal tibial apophysis; E embolus; EC embolus column; EM embolic membrane; EP embolus proper; FiG Fickert’s gland; LC lamella characteristica; P paracymbium; PH pit hook; PHS pit hook sclerite; PTP proximal tibial process; R radix; S1 upper chamber of spermatheca; S2 lower chamber of spermatheca; ST stretcher; TA terminal apophysis; TH thumb of embolus.

**Figure 3.**
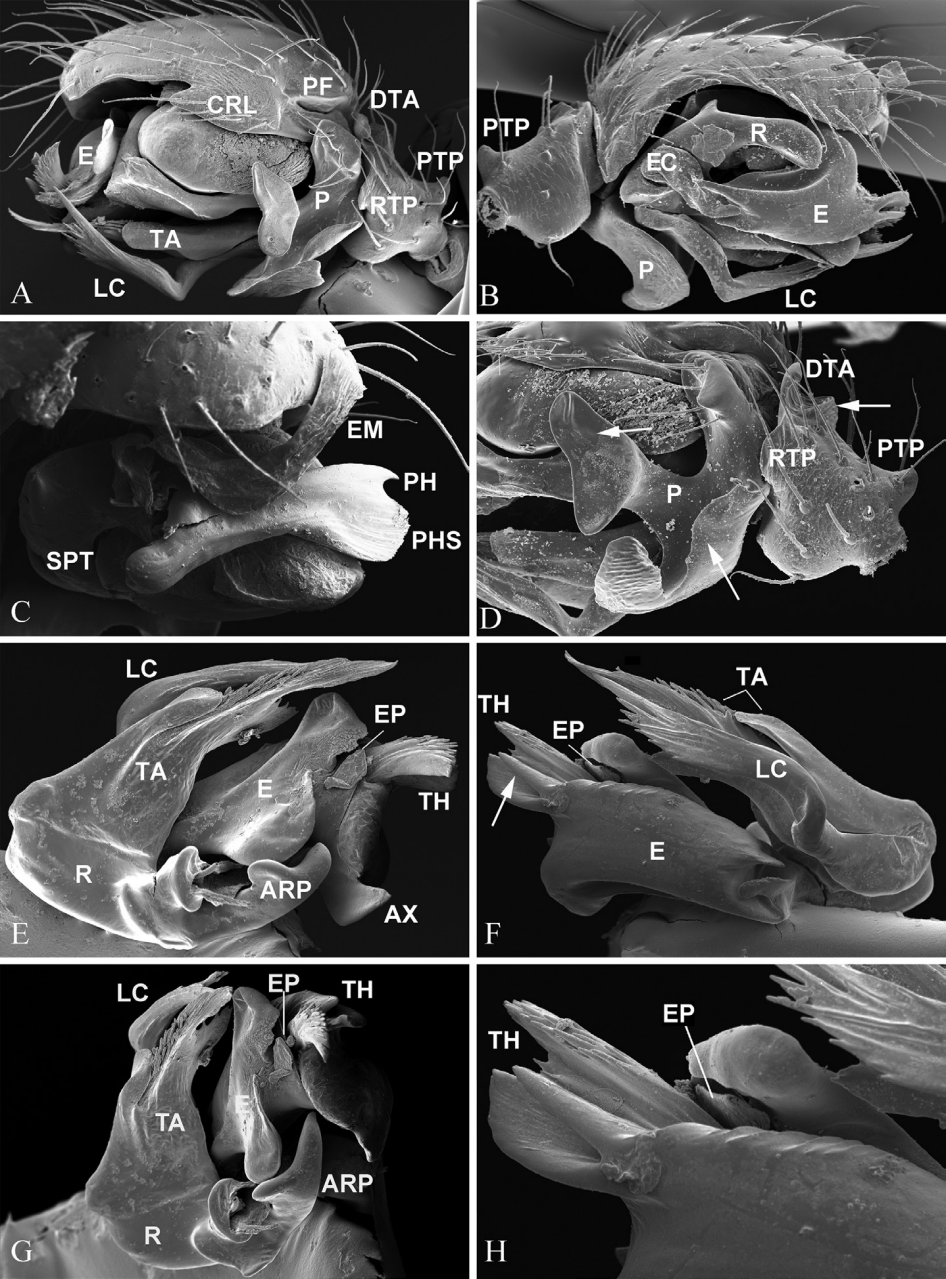
*Nippononeta alpina* comb. n., male palp. **A** retrolateral **B** prolateral **C** prolateral with embolic division removed **D** detail of A, arrows indicate serrated surface of DTA (upper right), median branch of paracymbium (upper left) and outer margin fold (lower) (**E–H**) embolic division **E** dorsal **F** ventral, arrow indicates embolic spine **G** anterior **H** detail of F, shows hided EP. Abbreviations: ARP anterior radical process; AX apex of embolus; CRL cymbial retrolateral lobe; DTA distal tibial apophysis; E embolus; EM embolic membrane; EP embolus proper; LC lamella characteristica; P paracymbium; PF posterior fold; PH pit hook; PHS pit hook sclerite; PTP proximal tibial process; R radix; SPT suprategulum; TA terminal apophysis; TH thumb of embolus.

**Figure 4.**
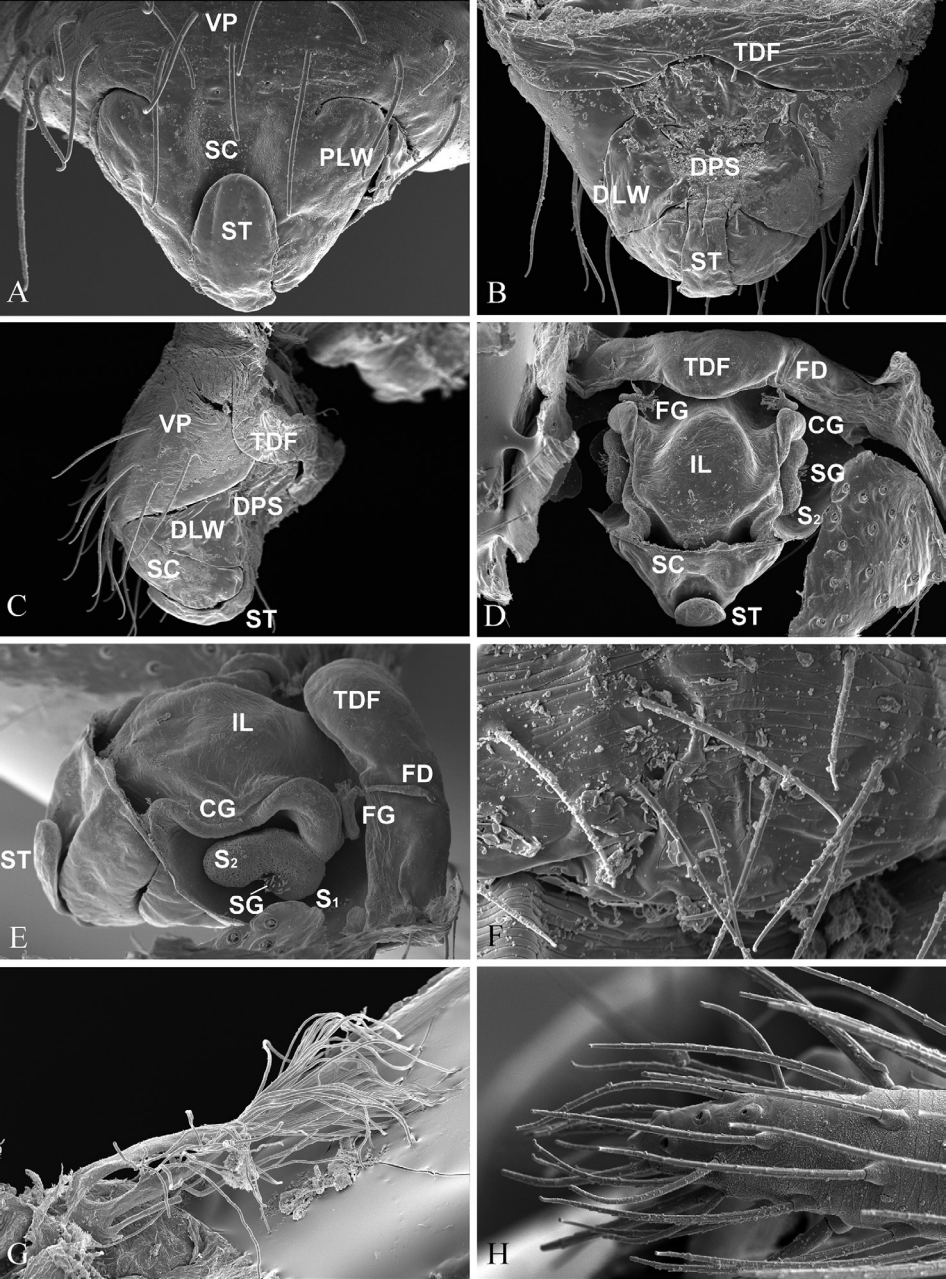
*Nippononeta alpina* comb. n. (**A–E**) epigynum **A** ventral **B** dorsal **C** lateral **D** ventral, cleared with ventral plate removed **E** lateral, cleared with ventral plate removed **F** male abdomen, ventral, shows epiandrous gland spigots absent **G** tracheal system, cleared **H** female palp, shows distal claw absent. Abbreviations: CG copulatory groove; DLW lateral wing on distal part of scape; DPS distal part of scape; FG fertilization groove; IL inner lobe; PLW lateral wing on proximal part of scape; S1 upper chamber of spermatheca; S2 lower chamber of spermatheca; SC scape; SG special gland; ST stretcher; TDF transversal dorsal fold; VP ventral plate.

The male of *Nippononeta alpina* comb. n. can be distinguished from all other *Nippononeta* species by the proximal tibial process (Fig. 2A) and the paracymbial median branch (Fig. 3D), both absent in other *Nippononeta* species. The female epigynum is distinguished by the epigynal cavity fully filled by the sigmoid folded scape, with a pair of lateral wings on the scape proximal part wrapping downward (Fig. 4A) and another pair of lateral wings on the scape distal part wrapping upward (Fig. 4B), while in most other *Nippononeta* species the epigynum usually diamond-shaped, with a dorsally opened epigynal cavity and a ventrally exposed scape (Yan et al. 2015: fig. 2A–B).

###### Description.

**Figure 5.**
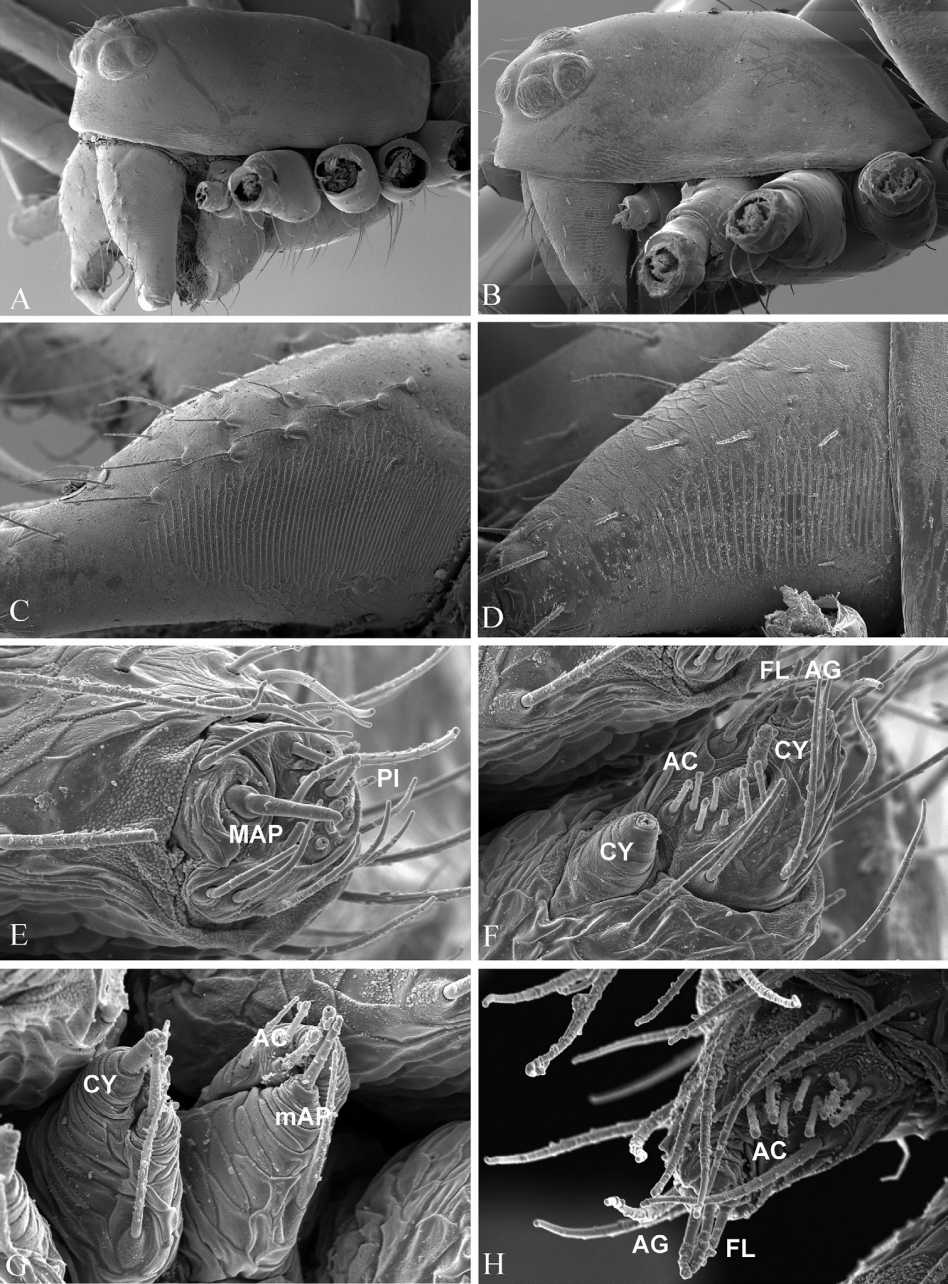
*Nippononeta alpina* comb. n. **A** male prosoma, lateral **B** female prosoma, lateral **C** male chelicerae, ectal **D** female chelicerae, ectal **E** female ALS **F** female PLS **G** female PMS **H** male PLS. Abbreviations: AC aciniform gland spigots; AG aggregate gland spigots; ALS anterior lateral spinneret; CY cylindrical gland spigot; FL flagelliform gland spigot; MAP major ampullate gland spigot; mAP minor ampullate gland spigot; PI piriform gland spigot; PLS posterior lateral spinneret; PMS posterior median spinneret.

Chelicerae of normal size, with narrower fang base and denser stridulatory ridges in the male than those in the female (Fig. 5C–D). Female palp without distal claw (Fig. 4H). Tracheal system having median trunk wider than the lateral pair, highly branched and extending into prosoma (Fig. 4G), tracheoles with taenidia. Epiandrous gland spigots absent in the male (Fig. 4F). Spinnerets (Fig. 4E–H): PLS in females having the mesal cylindrical gland spigot base enlarged (Fig. 5F), the triplet formed by one flagelliform and two aggregate gland spigots presented in the male PLS (Fig. 5H). For other somatic features, see description for the genus by Eskov (1992).

*Male palp* (Figs 2A–F, 3). Tibia short, as long as wide, with three apophyses: one distal, one retrolateral, one proximal; distal tibia apophysis with serrated surface. Cymbium with small retrolateral lobe and proximal fold above paracymbial base. Paracymbium U-shaped, distal arm shorter than proximal one, with well-developed median branch and outer margin fold. Distal suprategular apophysis modified as pit hook with hook sclerite. Embolic membrane furnished with many papillae. Embolic division: boat-shaped radix with ear-like anterior process. Embolus extremely complex, modified with multiple free ends; embolus proper covered by one of embolic sclerites; embolus thumb modified as spine-like projections; and apex triangular; Fickert’s gland located within embolus. Lamella characteristica unbranched, sigmoid ribbon-like in ventral view, with thread-like projections distally. Terminal apophysis divided into two parts: the posterior strongly sclerotized with a rounded end, the anterior membranous part with thread-like projections distally.

*Epigynum* (Figs 2G–K, 4A–E). Epigynal plate protruding out, with wide epigynal basal part. Median plate absent on dorsal surface, but the tegument of epigynal basal part forming transverse dorsal fold. Epigynal cavity fully filled by sigmoid folded scape, covered by a pair of lateral wings on scape proximal part wrapping downward, and another pair of lateral wings on scape distal part wrapping upward; stretcher lifting up. Copulatory tracts in groove state; fertilization tracts changing from groove to duct state and extending towards epigastric furrow.

###### Remarks.

*Nippononeta alpina* comb. n. originally was placed in the genus *Macrargus* Dahl, 1886, whose type species *Macrargus rufus* Wider, 1834 has a typical haplotracheate system (Blest 1976). In addition to tracheal characters, the genitalia of *Nippononeta alpina* are of a different type from that of *Macrargus rufus* (see descriptions by Saaristo in Marusik and Koponen 2008, Millidge 1977, 1984, Gnelitsa and Koponen 2010). Some genital characters of *Nippononeta alpina* are consistent with the putative synapomorphies for the genus *Nippononeta* and for the “desmitracheate micronetines” clade discussed bellow, but not shared by *Macrargus rufus*.

In addition, *Micrargus* is masculine in gender, while *Nippononeta* is feminine. As *Macrargus alpinus* is being transferred to *Nippononeta*, the species name has to be changed to *alpina*. However, Ono and Saito (2001) already described a species also named as *Nippononeta alpina* Ono & Saito, 2001, which is not a junior synonym of *Macrargus alpinus*. Therefore we propose a replacement name as *onoi*, after one author’s name, for the species of Ono and Saito to avoid homonymy.

## Discussion

Our results show that all desmitracheate “micronetines” form a monophyly, and *Macrargus alpinus* falls into the *Nippononeta* clade, distantly related to *Macrargus rufus* (Fig. 1). These suggest a new generic placement for this species, *Nippononeta alpina* comb. n. The two “micronetine” species, *Nippononeta alpina* and *Macrargus rufus* grouped into clade B is consistent with the putative synapomorphies based on somatic characters proposed for the seven-clade division (Wang et al. 2015). It is also in accord with the tracheal characters to group *Nippononeta alpina* with other desmitracheate “micronetines”, rather than with the haplotracheate genus *Macrargus*.

According to Wang et al. (2015) and Yan et al. (2015), some putative synapomorphies based on genital characters support the relationships among the desmitracheate “micronetines” and other linyphiids. The presence of a median plate is a synapomorphy for all linyphiids, but is secondarily lost in *Helophora*, *Nippononeta*, *Agyneta* and *Mesasigone*, as well as in *Nippononeta alpina* (Fig. 4B). Therefore, the absence of a median plate as a putative synapomorphy supports the sister relationship between the “desmitracheate micronetines” clade and *Helophora* (Fig. 1). The highly branched median pair of the tracheae, the protruding epigynum modified into a scape and epigynal cavity (Fig. 4A–D), and male palp with complex embolus (Fig. 1E) are putative synapomorphies for the former (Fig. 4G), while the moderately branched (Arnedo et al. 2009), the protruding epigynal plate without forming a scape and epigynal cavity (Tu and Hormiga 2010: fig. 7), and by the flagelliform embolus of the male palp (Chamberlin and Ivie 1933: fig. 85, Tao et al. 1995: fig. 69) are putative synapomorphies for the latter. Furthermore, the monophyly of the clade including *Nippononeta* and *Agyneta* (unknown for *Mesasigone*) is supported by four putative synapomorphies: by the presence of a serrated distal tibial apophysis and a retrolateral tibial process on the male palp (Fig. 3D, see also Yan et al. 2015: fig. 1D); by the dorsally opened epigynal cavity resulting from a secondary loss of the median plate (Fig. 4B, see also Yan et al. 2015: fig. 2B); and by the fertilization tracts changing from groove to duct state (Fig. 4E, see also Tu and Hormiga 2010: fig. 6c, Yan et al. 2015: fig. 5D).

The monophyly of the *Nippononeta* clade is supported by the following four putative synapomorphies: the pointed apophysis on the paracymbial proximal arm (Fig. 3D, Yan et al. 2015: fig. 1D); the embolus thumb modified into spine-like projections on the male palp (Fig. 3H, Yan et al. 2015: fig. 1G); and the presence of a transverse dorsal fold and finger-like stretcher (Fig. 1K, Yan et al. 2015: fig. 2A). Furthermore, the new placement of *Nippononeta alpina* implies that several putative synapomorphies previously proposed for *Nippononeta* by Yan et al. (2015) have to be reviewed: the narrowed epigynal basal part and the expanded lateral epigynal shoulder (Yan et al. 2015: fig. 2A–D) are not present in *Nippononeta alpina*. Meanwhile, *Nippononeta alpina* also has some apomorphies not shared with other *Nippononeta* species: the two pairs of scape lateral wings and the uplifting stretcher (Fig. 4A–D).

The monophyly of *Agyneta* is not supported and its relationships with other desmitracheate “micronetines” remain unresolved. Nevertheless, morphological studies show that *Agyneta* species are easily distinguished by some genital characters: e.g. the presence of a conical cymbial elevation and a sickle-shaped embolus with a large thumb, and the scaped epigynum with a pair of well-developed lateral lobes (see the review of Dupérré 2013). The uncertain phylogenetic relationship between *Agyneta* and other desmitracheate “micronetines” is largely due to a limited species-level sampling for such a diverse group. Clearly, more comprehensive sampling is needed for future studies.

## Supplementary Material

XML Treatment for Nippononeta

XML Treatment for Nippononeta alpina

## Supplementary materials

Supplementary material 1 GenBank accession numbers Data type: molecular data This dataset is made available under the Open Database License (http://opendatacommons.org/licenses/odbl/1.0/). The Open Database License (ODbL) is a license agreement intended to allow users to freely share, modify, and use this Dataset while maintaining this same freedom for others, provided that the original source and author(s) are credited.Mengdie Bao, Zishang Bai, Lihong Tu
