# Supplementary material for: On a desmitracheate “micronetine” Nippononeta alpina (Li & Zhu, 1993), comb. n. (Araneae, Linyphiidae)
Source: Zookeys. 2017 Jan 13;(645):133–46. doi: 10.3897/zookeys.645.10685 (PMC5299228; doi:10.3897/zookeys.645.10685)
Supplement: Supplementary material 1 — GenBank accession numbers [file zookeys-645-133-s001.doc]

Table S1. GenBank accession numbers.

| Family | Subfamily | Species | 16s | 18s | 28s | COI | Voucher |
| --- | --- | --- | --- | --- | --- | --- | --- |
| Linyphiidae | Micronetinae | *Nippononeta alpina* | KY318509 | KY318510 | KY318511 | KY318512 | CNU |
| Linyphiidae | Micronetinae | *Nippononeta kantonis* | GU338634 | GU338471 | GU338530 | GU338693 | CNU |
